# Pulvinar Modulates Synchrony across Visual Cortical Areas

**DOI:** 10.3390/vision4020022

**Published:** 2020-04-10

**Authors:** Nelson Cortes, Bruno O. F. de Souza, Christian Casanova

**Affiliations:** School of Optometry, Université de Montréal, CP 6128 succursale centre-ville, Montreal, QC H3C 3J7, Canada; b.oliveira.ferreira.de.souza@umontreal.ca (B.O.F.d.S.); christian.casanova@umontreal.ca (C.C.)

**Keywords:** cat, lateral posterior nucleus, reversible inactivation, transthalamic pathways, ventral stream, neuronal synchronization, Granger causality

## Abstract

The cortical visual hierarchy communicates in different oscillatory ranges. While gamma waves influence the feedforward processing, alpha oscillations travel in the feedback direction. Little is known how this oscillatory cortical communication depends on an alternative route that involves the pulvinar nucleus of the thalamus. We investigated whether the oscillatory coupling between the primary visual cortex (area 17) and area 21a depends on the transthalamic pathway involving the pulvinar in cats. To that end, visual evoked responses were recorded in areas 17 and 21a before, during and after inactivation of the pulvinar. Local field potentials were analyzed with Wavelet and Granger causality tools to determine the oscillatory coupling between layers. The results indicate that cortical oscillatory activity was enhanced during pulvinar inactivation, in particular for area 21a. In area 17, alpha band responses were represented in layers II/III. In area 21a, gamma oscillations, except for layer I, were significantly increased, especially in layer IV. Granger causality showed that the pulvinar modulated the oscillatory information between areas 17 and 21a in gamma and alpha bands for the feedforward and feedback processing, respectively. Together, these findings indicate that the pulvinar is involved in the mechanisms underlying oscillatory communication along the visual cortex.

## 1. Introduction

Most studies investigating the neuronal basis of vision consider cortical–cortical connections as the main pathways for information flow and processing yielding perception. In recent years, however, this corticocentric view of information processing has been challenged by considering the role of indirect pathways involving the pulvinar nucleus [1]. The pulvinar, the largest extrageniculate thalamic nucleus in mammals, provides another route of communication between cortical visual areas, via cortico-thalamo-cortical projections [2]. In brief, the pulvinar receives direct connections from the primary visual cortex and extrastriate visual cortical areas and, in turn, projects back to these areas [3,4,5]. Thus, the pulvinar is at the center of several cortical transthalamic pathways and consequently it is not surprising to learn that it is involved in several visual processes such as attention [6], visual salience [7], emotion perception [8] and contrast sensitivity [9]. 

It is well established that visual cortical areas are organized in a hierarchical manner where visual information travels from low to high levels (feedforward pathway), as well as from high to low levels (feedback pathway) [10]. These anatomical pathways appear to present a specific oscillatory-band range. During visual processing, gamma oscillations are found in the feedforward route, whereas slow oscillations, alpha/beta waves, are observed in the feedback direction [11]. In fact, the origin of these oscillations within a cortical column follows in parallel the anatomical connectivity of the feedforward and feedback pathways: gamma waves appear in layer 4 and propagate to superficial layers, while slow oscillations start in superficial and deep layers [12]. Thus, in addition to the anatomical hierarchy of the visual cortex, a functional hierarchy based on the neuronal synchronization appears as a possible marker of visual processing between cortical areas [13]. Accordingly, the way in which cortical areas are dynamically coordinated can be investigated by analyzing the oscillatory responses from neuronal populations. 

Previous studies have suggested that the pulvinar may be involved in the control of oscillations in the visual cortex. For instance, the inactivation of the pulvinar desynchronized the coupling between pairs of electrophysiological recordings in early visual cortical areas (areas 17 or 18) of anesthetized cats [14,15]. More recently, a few studies in awake primates investigated the pulvinar contribution to cortical communication between visual areas located at higher levels of the cortical hierarchy (from area V4 and beyond) [16,17]. These studies revealed that the pulvinar synchronized activity between interconnected cortical areas in the alpha range, possibly due to the top-down feedback modulation caused by the attentional-related task. This is supported by the increase in alpha waves, and decrease in gamma-band coherence, between V4 and the inferotemporal (IT) cortex during pulvinar lesions [6]. Together, these findings suggest that the pulvinar participates in the functional coordination of cortical interactions. However, its impact on the laminar source of oscillations and on the intracortical circuitry remains largely unknown. 

In the present study, we investigated the role of the pulvinar on the synchrony of cortical oscillations across two visual cortical areas located at distinct hierarchical levels. Multi-unit activity (MUA) and local field potentials (LFPs) were recorded in areas 17 and 21a of cats before and after the reversible inactivation of the pulvinar. These cortical areas are considered as homologues of areas V1 and V4 of primates, respectively [18] and present distinct patterns of thalamic connectivity [19,20,21]. The present findings show that the pulvinar inactivation yielded changes in the low (alpha) and high (gamma) bands of cortical oscillations in both visual areas. Furthermore, these oscillatory responses in cortical areas were layer dependent. In addition, Granger causality analysis suggests that during visual stimulation, the pulvinar could modulate the feedforward gamma band from areas 17 to 21a. Interestingly, feedback alpha waves from area 21a to area 17 arose even before the visual stimulation. Together, these findings indicate that the pulvinar plays a role in the directionality of oscillatory feedforward and feedback processing along the visual cortex.

## 2. Material and Methods

### 2.1. Animals and Surgery

Experiments were performed on two normal female adult cats (2.5–3.5 kg). All surgical and experimental procedures were performed according to the guidelines of the Canadian Council on Animal Care and were approved by the Ethics Committee of the University of Montreal (CDEA 19-008). Atropine (0.1 mg/kg) and acepromazine (Atravet, 1 mg/kg) were administered subcutaneously to reduce parasympathetic effects of anesthesia and to provoke sedation, respectively. Anesthesia was induced with 3.5% Isoflurane in a 50:50 (*v*/*v*) gas mixture of O_2_ and N_2_O. Isoflurane concentration was maintained at 1.5% throughout surgical procedures. A tracheotomy was performed, and animals were immobilized using an intravenous bolus injection of 2% gallamine triethiodide. Then, animals were artificially ventilated and a 1:1 (*v*/*v*) solution of 2% gallamine triethiodide (10 mg/kg/h) in 5% of dextrose in lactated Ringer’s was continuously administered intravenously to maintain muscular relaxation and to provide nutrition and electrolytes. Expired level of CO_2_ was maintained between 35 and 40 mmHg by adjusting the tidal volume and respiratory rate.

The temperature was maintained at 37 °C by means of a feedback-controlled heated blanket. Heart rate was continuously monitored during the experiment. Local anesthetic (lidocaine hydrochloride 2%) was used in all incisions and pressure points. Dexamethasone (4 mg, i.m.) was administered every 12 h in order to avoid cortical swelling. Pupils were dilated using atropine (Mydriacyl) and nictitating membranes were retracted using phenylephrine (Midfrin). Rigid contact lenses with the appropriate power were used to correct the eyes’ refraction and eye lubricants were used to avoid corneal dehydration. Three craniotomies were performed in order to gain access to the LP nucleus (5–8A; 3–7L, Horsley–Clarke coordinates) and to cortical areas 17 (4–8P; 0.5–2L) and 21a (2–6P; 7–11L). Small durectomies were performed for each electrode penetration, and a 2% saline agar solution was applied over the exposed cortex to increase recordings’ stability and to avoid the drying of the cortical surface.

### 2.2. Visual Stimuli

Visual stimuli were generated using the VPixx© software (VPixx Technologies Inc., St-Bruno, QC, Canada) driven by a Macintosh 2.66 GHz computer. Stimuli were rear-projected by an EIKI LC-WB200 projector (80 Hz, 1208 × 768-pixel resolution) onto a diffused translucent screen (Da-Lite© screen). The screen was located at a viewing distance of 57 cm and covering 104° × 79° of visual angle with a mean luminance of 25 cd/m^2^. Stimuli consisted of drifting sinusoidal gratings with spatial and temporal frequencies set at 0.3 cpd and 3 Hz for all stimuli, respectively. The spatial and temporal frequencies were inside the response range of neurons from both areas 17 [22,23] and 21a [24,25,26]. Trials were fully randomized, and each stimulus was presented 15 times. To characterize oscillatory responses, visual stimuli were presented at 100% contrast. In the current study, responses to all directions were considered. Each trial lasted 3 s. During the first 0.5 s, a mean luminance gray screen was presented (blank). In the next 2 s, the visual stimulus was presented. Another 0.5 s of blank was presented at the end of the trial. 

### 2.3. Electrophysiological Recordings and Signal Preprocessing

During recording sessions, the anesthesia was changed to Halothane (0.5–0.8%). This was done to maintain the cortical responsiveness since it has been shown that isoflurane yields a strong depression of visual responses [27]. Neural activity in areas 17 and 21a was recorded simultaneously using 32-channel linear probes (~1 MΩ, 1 × 32–6mm–100–177, Neuronexus), with contact points spaced 100 μm apart. 

Prior to the insertion, the probes were covered with a fluorescent dye (DiI) allowing the histologic assessment of the electrode position in the cortex. Electrophysiological signals were acquired at 30 kHz and band pass filtered between 1 and 7500 Hz using an open-source system (Open-Ephys platform [28]). Local field potential (LFP) and multi-unit activity (MUA) were obtained from the same electrode. LFP was recorded by low-pass filtering (< 200 Hz) the raw data. 

MUA was measured by first band-pass filtering (500–5000 Hz), then full wave rectified, and finally low-pass filtering (200 Hz) the digitized signal. The MUA is the representation of the envelope of the neuronal response at high frequency [29], and it was used to obtain an instantaneous and dynamic measure of the population firing rate. MUA responses were calculated by integrating the MUA (MUAe) over the visual stimulation period. 

### 2.4. Signal Processing

A preliminary Fourier analysis was performed on LFPs to explore oscillatory cortical responses [30]. Only the power spectrum, *S*, was obtained by multiplying the fast Fourier transform (FFT) output *X* with its complex conjugate *X’*, and normalizing by the number of data points *N* as

(1) $$S=\frac{X\cdot X^{\prime }}{N}.$$

Wavelet analysis was used to determine the amplitude of the signal per time and frequency. To that end, LFPs were convolved with complex Morlet wavelets [31]. Complex Morlet wavelets, *w*, are defined as

(2) $$w\left(t,f\right)=A\cdot exp\left(-t^{2}/2\sigma_{t}^{2}\right)\cdot exp\left(2\pi ift\right),$$

where *A* is the normalization factor, $A={\left(\sigma_{t}\sqrt{\pi }\right)}^{-1/2}$, *t* the time, *f* the center frequency of the wavelet, and $\sigma_{t}$ the standard deviation of the Gaussian taper, using $\sigma_{t}=1/\left(2\pi f\right)$. The result of the average total power was transformed to the decibel scale by normalizing the responses with the average of the pre-stimulus baseline (−500 to 0 ms) as

(3) $$dB_{tf}=10log\left(\frac{activity_{tf}}{mean{\left(baseline\right)}_{f}}\right),$$

where *t* and *f* are the time and frequency points. The baseline average has no *t*, showing that the whole sequence of time points within a frequency was normalized by the pre-stimulus period. The decibel conversion assures that the frequency-band-specific power is the change in power relative to the baseline rather than to the absolute level of power, and so, any spurious constant activity (e.g., background activity) over time is removed. 

To obtain oscillatory responses, alpha (7.5 to 12.5 Hz) and gamma (30 to 80 Hz) bands were selected from the decibel-corrected power data, and the average responses across bands were calculated. The area under the curve (AUC) of the oscillatory bands was collected, and then normalized over the period of visual stimulation. The change in decibels (dB) over time from the baseline was reported. To quantify the effect of thalamic inactivation, a percentage variation (%Var) was used. This was calculated by normalizing the distribution of oscillatory responses for the periods during and after the inactivation by the distribution obtained before the inactivation (*x^Ctr^*), as

(4) $$Var\left(Ctr\right)=\frac{x-x_{mean}^{ctr}}{x_{max}^{ctr}-x_{min}^{ctr}}\times 100,$$

where *x* is the distribution of dB over time. The results were then multiplied by 100 to obtain %Var as a function of the control. 

To compute Granger causality, the MVGC toolbox [32] was used with a model order *m* = 10 (50 ms) [33]. Windows of 500 ms were used to calculate GC for each experimental trial. This window duration minimized possible nonstationary variations of neuronal responses [31]. Granger causality between layers of areas 17 and 21a was calculated after histological confirmation of electrodes positions. Significant differences were quantified by bootstrapping (See Statistical Analysis).

### 2.5. Thalamic Inactivation

The lateral subdivision of the LP nucleus (LPl) was pharmacologically inactivated by the intracerebral injection of a solution of 20 mM gamma aminobutyric acid (GABA) stained with Chicago Sky Blue (0.5%) for the histologic assessment of the location and extent of the injection. The GABA solution was injected using a custom-made injectrode [34]. First, the solution was injected at a rate of 80 nL/min until the inhibition of the neuronal activity was achieved. A successful inactivation was characterized by the silencing of the local multiunit activity recorded through the injectrode. Subsequently, the injection rate was reduced to 20–40 nL/min to silence neural activity throughout the testing period. The approximate total volume of GABA solution injected was 1 μL. The local neuronal activity was continuously monitored and a recovery from inhibition was observed about 30–45 min after the completion of the GABA injection. Cortical responses were recorded before (control, Ctr), during (injection, Inj), and after (recovery, Rec) GABA injection in the thalamus (Figure S1).

### 2.6. Histology

At the end of the experiment, animals were euthanized with an intravenous injection of sodium pentobarbital (Euthanyl, 110 mg/Kg). Animals were transcardially perfused with a phosphate buffer solution (0.1 M, pH 7.4) followed by a fixative (Paraformaldehyde 4%). Brain tissue was cryoprotected using sucrose solutions at different concentrations (10%–30%), frozen and stored at −80 °C. Then, 40 μm coronal sections were obtained and subsequently stained. LP subdivisions were revealed using acetylcholinesterase staining [35]. The Chicago Sky Blue staining was used to locate the injection sites and to provide a rough estimation of the extent of the GABA diffusion in the thalamus. Cortical layers were identified using DAPI and the DiI fluorescence signal was used to reconstruct the electrode position. Furthermore, immunostaining of nonphosphorylated neurofilament protein was used to confirm the position of recordings in area 21a [36].

### 2.7. Selection of Oscillatory Signals Based on the Influence of MUA and the Stability across Conditions

For each LFP, two selection processes were performed to analyze the amplitude of the wavelet signal from the control, injection, and recovery conditions. This selection criterion used MUAe and oscillatory responses from the same electrode. The first step consisted of selecting MUAe that increased during the LPl inactivation. So, MUAe before the inactivation was less than during inactivation (Inj-Ctr > 0). Then, alpha and gamma waves from these increased MUAe, same electrode, were selected. This first selection was based on the results obtained in awake animals performing an attentional task [30]. Here, alpha and gamma waves in V1 were negatively and positively correlated with the MUAe, respectively. In the next step, only the oscillatory activity was used. Here, alpha and gamma waves during the LPl inactivation should be lower than the activity recorded in the recovery period. So, oscillatory activity decreased after the inactivation period (Inj-Rec > 0). This second action ensured that only the oscillatory activity that returned toward control conditions (i.e., partial recovery) or was fully reversed was considered. These two criteria allow minimizing cortical fluctuations and non-stationary processes after the inactivation of the LPl. 

### 2.8. Statistical Analysis

**Figure 1:**
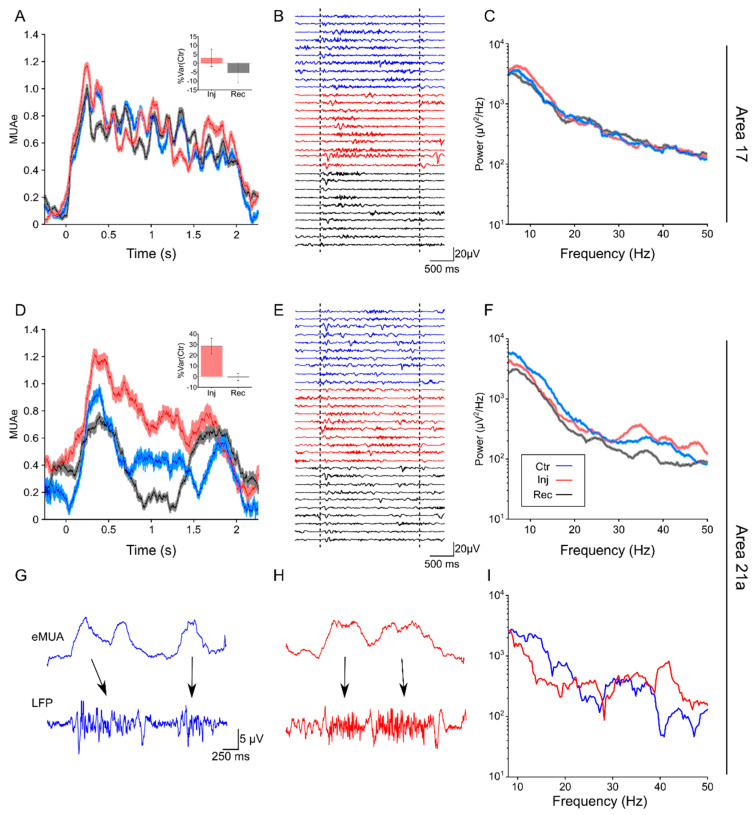
Oscillatory cortical responses during the inactivation of the LPl. Representative examples of simultaneous electrophysiological recordings in areas 17 and 21a, evoked by drifting gratings during control (Ctr, blue trace), injection (Inj, red trace) and recovery (Rec, black trace), conditions. (**A**) Average MUAe, (**B**) ten local field potentials (LFP) samples, and (**C**) average LFP power spectra are responses from an electrode placed in layer II/III of area 17 (Animal 2). The inset in A shows the %Var(Ctr) for Inj and Rec periods. (**D**) Average MUAe, (**E**) ten LFP samples, and (**F**) average LFP power spectra are responses from another electrode placed in layer IV of area 21a (Animal 2). The inset in A shows the %Var(Ctr) for Inj and Rec periods. (**G**) and (**H**) are cortical oscillatory responses that come from the same electrode shown in panels D, E, and F (layer IV, area 21a). Panels (**G**) and (**H**) show MUAe and LFP before and during the injection of GABA in the pulvinar, respectively. The arrows indicate the co-activation between the MUAe and the oscillatory activity in the LFPs. (**I**) LFP power spectrum evoked by the visual stimulation in Ctr and Inj conditions. Note that oscillatory response around the gamma band (40 Hz) is facilitated by the inactivation of the LPl (red line, panels (**H**) and (**I**). In panels (**A**), (**C**), (**D**) and (**F**), shadow areas represented the bootstrapping of random averages (1000 repetitions), to obtain a confidence interval at a threshold P = 0.05.

All data were also analyzed using Matlab functions (MATLAB 2018; Math Works Inc., Natick, MA, USA). Data are expressed as mean ± s.e.m, unless otherwise stated. The nonparametric test Kruskal–Wallis was used to compare differences of cortical responses during three conditions: before, during and after the injection of GABA in LPl. When the test revealed significant differences, pairwise data comparison was performed using Wilcoxon rank-sum tests and *p*-values were adjusted with Bonferroni corrections. Correlation analysis was performed and Pearson’s correlation coefficient (r) and significance values were shown. Bootstrapping was used to calculate the standard error of the mean (s.e.m) (1000 repetitions) of averaged laminar signals (for Figure 1A,C,D,F; and Figure 6B2,B3). The distribution of repetitions enables us to determine confidence intervals, and *p*-values between layers (the 2.5 and 99.75 percentiles for a *p*-value of 0.05). 

## 3. Results

In the current study, 593 visual evoked responses (VERs) were recorded in areas 17 (~45%, 268 VERs) and 21a (~55%, 325 VERs). VERs were analyzed before, during and after the pharmacological inactivation of the cat LPl. The LPl subdivision was targeted because is the only subdivision of the cat pulvinar that is connected reciprocally to both areas 17 and 21a [20,36]. Cortical local field potentials (LFPs) and multi-unit activity (MUA) were acquired and the laminar profile of oscillatory activity in each cortical area was characterized. The impact of LPl inactivation on the cortical oscillations was assessed by the analysis of changes in amplitude of LFP’s alpha and gamma bands. In addition, Granger causality was used to assess the strength of oscillatory coupling between layers of areas 17 and 21a when the LPl was inactivated.

### 3.1. LPl Inactivation Changes Oscillatory Responses in Areas 17 and 21a

Silencing the LPl increased the amplitude of oscillatory signals in areas 17 and 21a. Figure 1 shows three representative examples of the impact of the LPl inactivation on oscillatory cortical responses. The thalamic inactivation yielded a very small increase in MUAe (%Var(Ctr) = ~3.6% ± 6.64%) in layer II/III of area 17 (panel A), with a moderate increase in amplitude of the LFP oscillatory signal (panel B). Here, cortical oscillatory activity enhanced was partial, showing an increased amplitude of the LFP power spectra mostly in the lower frequency range (panel C). Panels D, E and F show the oscillatory responses in layer IV of area 21a. Both the MUA (panel D) and LFP (panels E and F) signals disclosed an enhancement of neuronal activity during LPl inactivation (%Var(Ctr) = ~28% ± 7.55), with power spectra showing larger changes at higher frequencies (Panel F and I). In addition, a co-activation of the MUA and the LFP was observed in some recordings for layer IV of area 21a (arrows on panels G and H). This co-activation before (panel G) and during (panel H) pulvinar inactivation showed similar oscillatory profiles. However, a more detailed oscillatory analysis (panel I, LFP power spectra) ratifies an enhancement of the gamma range during the LPl inactivation. These results indicate that the LPl inactivation modulated the cortical activity with distinct effects on the spectral profile of oscillations in areas 17 and 21a. While layers II/III of area 17 had a slight increase in alpha-band frequency, layer IV in area 21a showed a substantial increase during the pulvinar inactivation. In order to quantify whether those effects were representative throughout the data, the amplitude of the oscillatory responses was computed using a wavelet analysis of the LFP signal.

**Figure 2:**
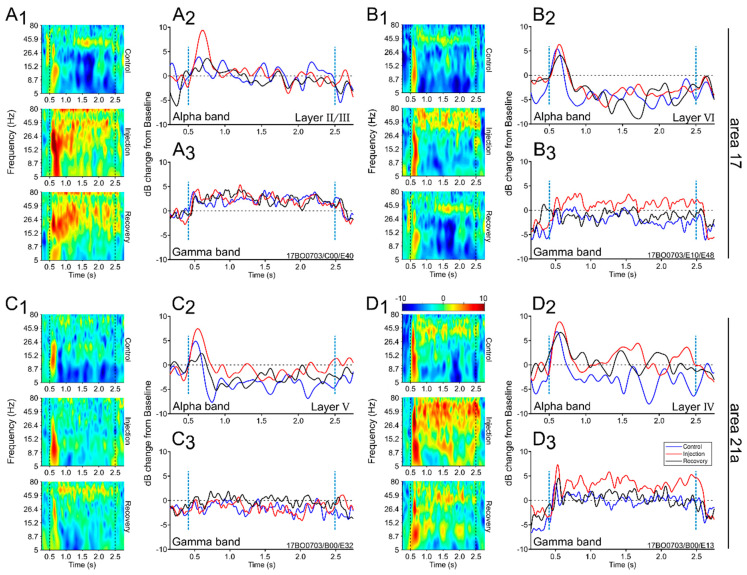
Oscillatory analysis for areas 17 and 21a recordings across experimental conditions. Wavelet analysis was used to calculate spectral estimates. (1) Time-frequency figures for representative samples. (2) Average time-frequency traces of alpha ranges. (3) Average time-frequency traces of gamma ranges for Ctr, Inj and Rec conditions. (**A**) Layer II/III recording from area 17. (**B**) Layer VI recording from area 17. (**C**) Layer V recording from area 21a. (**D**) Layer IV recording from area 21a.

The oscillatory changes that occurred throughout the visual stimulation and experimental conditions may have violated the stationarity of the neuronal responses. To overcome such a problem, Morlet wavelet convolution was used. This method assumes that the data are stationary within relatively short periods of time [30]. The second benefit of wavelet convolution is the ability to detect changes in frequency structure over time (time-frequency analysis). Thus, the Morlet wavelet convolution provides an appropriate tool to detect and analyze non-stationary signals [31]. Figure 2 depicts four representative examples of the time-frequency analysis of LFPs undertaken using the wavelet algorithm. Each panel shows the LFP spectrogram and the time course of changes in the signal amplitudes from the baseline (see methods) of alpha and gamma ranges from cortical layers of area 17 (Figure 2A,B) and 21a (Figure 2C,D). The responses were similar to the representative cases shown in Figure 1C,F.

In panel A1, a significant rise in low oscillatory responses was seen in layer II/III of area 17 during LPl inactivation. The increased alpha band amplitude was restricted to the first 500 ms after stimulus onset (Figure 2A2). On the other hand, the LPl inactivation had no visible impact on the gamma band since there was an overall increase in activity during the stimulus presentation in all conditions (panel A3). Another visual-responsive oscillatory pattern was observed in layer VI of area 17 (Figure 2B). Here, low-wave responses were increased across all conditions (panel B2) and, as in Figure 2A, the changes in the alpha range were constrained to the 500 ms after the stimulus onset. For this layer, the LPl inactivation yielded an increase in the amplitude at higher frequencies (panel B1). In contrast to the alpha waves (panel B2), changes in the gamma range were sustained during stimulus presentation (panel B3). The increased gamma oscillations were observed in different magnitudes throughout our dataset. One possible explanation is that gamma oscillations are facilitated by the halothane anesthesia, as previously reported in the visual cortex of rodents [37].

In area 21a, the layers that were activated due to the inactivation of the LPl were different. Layer V showed an increase in the low oscillations evoked during thalamic inactivation while high-frequency oscillations were poorly represented (panel C1). The alpha waves were highly evoked at the beginning of the stimulation (0–500 ms) (panel C2), whereas the gamma-frequency band had a weak signature (panel C3). For layer IV of the same area, high-frequency bands increased during the LPl inactivation (panel D). Indeed, a strong gamma-frequency band was evoked during the visual stimulation (panel D3) while alpha-band responses increased during the first 500 ms interval after the stimulus onset (panel D2). This transient increase in alpha waves was similar for the three experimental periods. 

**Figure 3:**
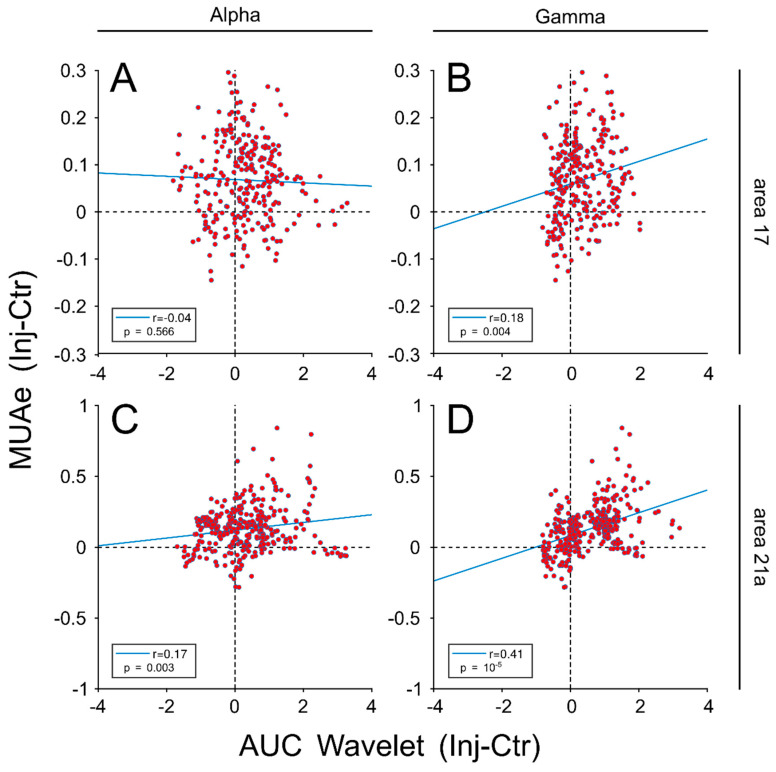
Influence of MUA activity on the oscillatory cortical responses of areas 17 and 21a. Area under the curve (AUC) of alpha- and gamma waves calculated by taking the amplitude of the wavelet analysis across the visual stimulation interval (Figure 2). The differences in the AUC between injection and control conditions for alpha- and gamma waves were correlated with the differences in the AUC for MUAs in the same periods. (**A**) Correlation for alpha band and MUA in area 17. (**B**) Correlation for gamma band and MUA in area 17. (**C**) Correlation for alpha band and MUA in area 21a. (**D**) Correlation for gamma band and MUA in area 21a.

One observation arising from the previous examples is that LFPs power signals increased when MUAe responses also increased (Figure 1G–I). For areas 17 and 21a, ~75% (n = 201) and ~79% (n = 257) of VERs exhibited an increase in MUA responses during injection periods, respectively. However, these variations were heterogeneous across cortical layers, in particular for area 17 (Table S1). To obtain a measure of variation over the visual stimulation interval, alpha- and gamma-frequency bands were determined by averaging the AUC from the selected oscillatory ranges from the time-frequency wavelet analysis. The next step was to correlate MUAe and alpha and gamma synchronization for the difference between injection and control periods. These variations are presented in Figure 3. While positive correlations for the gamma frequency were observed on electrodes located in areas 17 (r = 0.18, *p*-value = 0.004, panel B) and 21a (r = 0.41, *p*-value < 0.001, panel D), alpha frequency correlated only for units located in area 17 (r = 0.17, *p*-value = 0.003) (panel C). A more detailed analysis for each cortical layer showed that the changes in gamma band oscillations were significant for layer VI (r = 0.27, *p*-values < 0.05) and partially for layer IV (r = 0.23, *p*-values = 0.06) in area 17, and highly significant for all layers in area 21a, except layer I (All r > 0.4 and *p*-values < 0.01) (Figure S2, Table S2). Consequently, given that the oscillatory responses were more represented during injection periods, units that had MUAe larger responses in injection periods were selected for the next set of analyses. 

**Figure 4:**
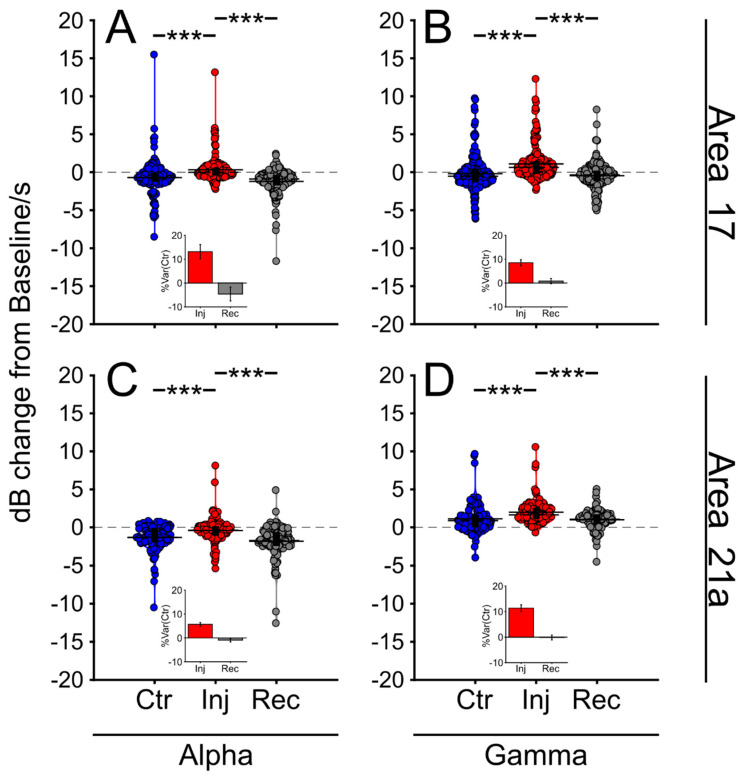
Global oscillatory cortical responses before (Ctr), during (Inj) and after (Rec) LPl inactivation. Comparison of the amplitude of alpha- (**A**) and gamma waves (**B**) across conditions for cortical signals in area 17. Comparison of the amplitude of alpha- (**C**) and gamma waves (**D**) across conditions for cortical signals in area 21a. Insets show the %Var(Ctr) of the Inj and Rec periods. *** P < 0.001.

Although MUAes and oscillatory responses were correlated, several cortical signals drifted after the inactivation of the LPl. Such fluctuations were avoided by selecting oscillatory responses that had larger AUCs in injection than in recovery periods. This process ensured that the analyzed signal was stable over time as it returned to control values. The result of this analysis is shown in Figure 4 (Table S3). Here, the amplitude of alpha and gamma waves were significantly higher during the injection period than during the control and recovery periods. The LPl inactivation yielded mostly an increase in the amplitude of alpha and gamma bands of the LPF signals recorded in areas 17 (Figure 4A,B) and 21a (Figure 4C,D). However, the %Var(Ctr) showed that the oscillatory activity varied little during the injection period. On average, the activity varied by ~10% (Figure 4, insets). Since all cortical layers were selected, this analysis could be masking differences across cortical layers (Table S4). Moreover, the LPl inactivation on areas 17 and 21a effects varied considerably throughout the cortical depth (Table S1). Thus, in the next section, the layer dependency of the thalamic inactivation was investigated by comparing the changes in the amplitude of alpha and gamma oscillations across the cortical layers.

### 3.2. LPl Inactivation Affected Heterogeneously Alpha- and Gamma-Frequency Band Responses across Cortical Layers in Areas 17 and 21a

**Figure 5:**
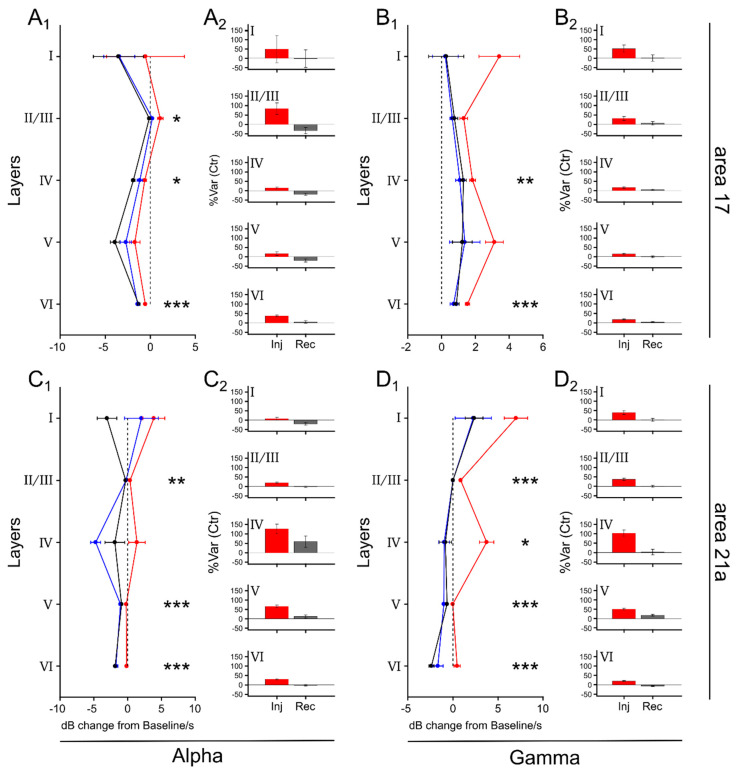
Effect of LPl inactivation in alpha- and gamma-band frequency for layers in areas 17 and 21a, respectively. (1) Values represent average relative LFP power spectra (dB change from the baseline/s). This parameter measures the amplitude of cortical oscillations in response to drifting gratings before (Ctrl, blue lines), during (Inj, red lines) and after (Rec, black lines) LP inactivation. (2) Var%(Ctr) across layers for periods during and after the thalamic inactivation. (**A**) and (**B**) alpha waves and gamma waves for in area 17, respectively. (**C**) and (**D**) alpha waves and gamma waves for in area 21a, respectively. Symbols in panels (**A1**), (**B1**), (**C1**) and, (**D1**) only are for significant differences between Ctrl and Inj conditions. * P < 0.05, ** P < 0.01, *** P < 0.001.

AUCs and %Var(Ctr) for alpha- and gamma-frequency responses were plotted as a function of cortical layers for areas 17 and 21a (Figure 5, and Tables S5 and S6). In panel A1, alpha responses in area 17 were mainly affected in layers II/III during LPl inactivation, showing only positive responses here. Significant differences between control and injection conditions were observed for layers II/III, IV and VI (Figure 5A1). The %Var(Ctr) during inactivation showed that the response in these layers increased in a ~84%, ~14% and ~37%, respectively (Figure 5A2). For gamma responses, only layers IV and VI showed significant differences between injection and control periods (Figure 5B1). For those layers, the %Var(Ctr) was ~16% (Layer IV) and ~17% (Layer VI) during the inactivation. In area 21a, all layers showed significant alpha-wave responses, but not layers I and IV (Figure 5C1), with the largest %Var(Ctr) for layer V (~66%) during LPl inactivation. For gamma waves, the layer IV showed most of the frequency-band changes during the thalamic inactivation (%Var(Ctr) of ~103%) (Figure 5D1,D2). Here, all layers showed a significant activity enhancement, except for layer I. In summary, the two cortical areas showed a similar pattern during the inactivation of LPl: high-frequency oscillations are more represented in layers IV and VI, whereas low-frequency bands are mostly seen in layer II/III.

### 3.3. LPl Inactivation Facilitates the Feedforward and Feedback Coupling between Areas 17 and 21a

The role of LPl on the directionality of the feedforward and feedback across cortical layers of areas 17 and 21a, was studied with Granger causality (GC). GC quantifies whether one time series is capable of forecasting the activity of another time series. High magnitudes of GC indicate a strong coupling between the two signals. Here, GC was used to predict the level of oscillatory coupling between layers of areas 17 and 21a. In this analysis, the same data from Figure 4 and Figure 5 were considered, but only the feedforward connection from layers II/III of area 17 to layer IV in area 21a, and the feedback connections from layer V of area 21a to layers II/III and V of area 17 were described. Anatomical feedback projections from layer VI of area 21a to layers II/III of area 17 have been previously reported [38], but, for low oscillations, the GC index showed no significant differences between periods before and during thalamic inactivation (Figure S3). 

**Figure 6:**
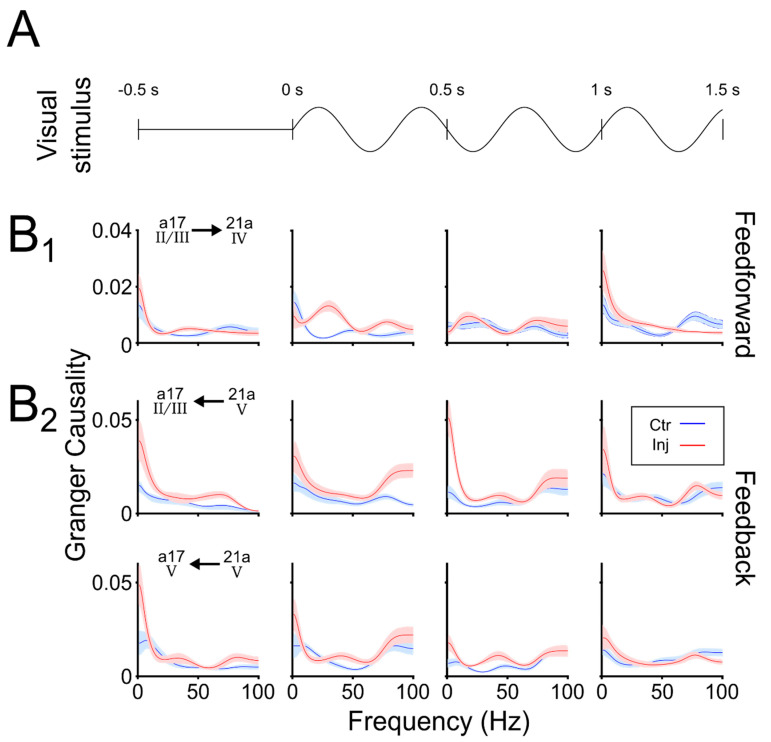
Granger causality of LFP signals for feedforward and feedback cortical coupling during the inactivation of the LPl. (**A**) Schematic 2D representation of the drifting grating stimulation over time. (**B1**) Granger causality of the feedforward and (**B2**) feedback coupling for windows of 500 ms before and during visual stimulation. Granger causality (GC) in the range of either alpha (6–13 Hz) or gamma band (25–40 Hz) synchronization. Shadow area represented the bootstrapping of random averages GC (1000 repetitions), to obtain a confidence interval at a threshold P = 0.05. Blue and red lines indicate before (Ctr) and during (Inj) pulvinar inactivation, respectively.

Figure 6 reveals that the thalamic inactivation changed the oscillatory response of feedforward and feedback coupling, depending on the visual stimulation onset (panel A). GC indices are shown in panel B for conditions before and during the LPl inactivation. We first analyzed the feedforward coupling (panel B1). Here, before the presentation of visual stimuli (−500–0 ms), the cortical coupling was similar for the two conditions. This situation changed during the beginning of the visual stimulation (0–500 ms). The GC index showed a peak at low-gamma activity (at 30 Hz). This 30 Hz was found only for the feedforward coupling and during this particular recording window. For the next temporal windows (500–1000 ms), the two conditions had similar oscillatory responses.

For the feedback coupling (panel B2), a different GC profile was depicted. Strong GC couplings at low-frequency bands were present before the presentation of the visual stimulation (−500–0 ms). In the next recording time window, when the visual stimulation was presented (0–500 ms), these low-frequency bands vanished. However, in the next visual stimulation window (500–1000 ms), the GC index for the inactivation condition increased again in the range of low-oscillatory waves. This profile of oscillatory activation was more evident for the feedback connections from area 21a reaching layers II/III than layer V of area 17. These findings suggest that during LPl inactivation, the GC coupling had a low gamma band enhancement from area 17 to area 21a (feedforward) evoked by the visual stimulation, and an alpha band increment from area 21a to area 17 (feedback) independent of the evoked visual responses.

## 4. Discussion 

In this study, we investigated whether the pulvinar can modulate the amplitude of intracortical visually driven neuronal oscillations. The inactivation of the LPl produced not only a global increase in cortical oscillations in the two areas but also a particular pattern of layer activation: high-frequency oscillations were more represented in layers IV and VI, whereas low-frequency bands were more present in layer II/III. In area 17, there was a moderate increase in alpha waves in layers II/III rather than in layers V or VI (see below). For area 21a, the gamma range had a more significant increase in layer IV. The GC analysis of the simultaneous multi-recordings in areas 17 and 21a showed a low gamma band enhancement from area 17 to area 21a (feedforward) and an alpha band increment from area 21a to area 17 (feedback). Interestingly, during the inactivation, the feedforward gamma band coupling increased at the beginning of the visual stimulation while the feedback alpha band coupling was greater before and in the middle of the visual presentation. Taken together, the propagation and amplification of these two rhythms along the hierarchy of the visual cortex co-exist during the LPl inactivation, but they seem to exclude each other since when one increases the other decreases and vice versa, as suggested by previous work [39]. 

### 4.1. Generation of Alpha-Wave Responses

Changes in alpha-frequency activity during the LPl inactivation were mild. In general, in the current study, alpha waves were more represented during the first second of the visual stimulation, decaying in amplitude after the initial interval. One explanation for this transient response is that the inactivation of LPl generated a stronger response in the feedforward pathway than the feedback pathway along the visual cortex. Then, as proposed at the end of the previous paragraph, the activity of the feedforward could suppress the action of the feedback pathway [39]. These feedforward influences are suggested by the robust anatomical pulvinar connections to layer I, for areas 17 and 18, and layer IV, for higher-order cortical areas [1]. In a previous study from our group [9], we investigated the impact of LP inactivation on the unitary response to contrast in areas 21a and 17 of the cats. The responses of neurons from area 21a were highly modulated by the thalamic inactivation, showing that the pulvinar has a stronger influence on the activity at higher cortical levels. Our results corroborate these findings and, in addition, show that this stronger influence is mainly conveyed through a feedforward pathway. Another explanation of the low level of alpha waves during LPl inactivation, is that the visual stimulation used in our experiments was inefficient to evoke such oscillatory activity. For example, the figure-ground segregation stimulation seems to evoke more prominent alpha-band frequencies than the full field drifting grating stimulus used here. In particular, because such alpha-band responses are strongly evoked on the background texture of the visual stimulation. Thus, alpha-band synchronization is stronger in orientations that are different to the figure configuration, which indicates a strong feedback processing [12]. This explains why a prominent oscillatory activity was evoked during the beginning of the visual stimulation (the first 500 ms after the stimulus onset) and not after. Another possibility for the transient alpha-wave response is that the duration of the stimulus was long enough to produce inhibition of the oscillatory activity by surround suppression [40]. This suppression results in the alpha band’s oscillatory activity not being as affected. It should not be forgotten, moreover, that the animals are under anesthesia, and this can vary the performance of alpha and gamma oscillatory activities considerably [37,41]. Further experiments considering complex visual stimuli are needed to increase our knowledge of alpha and gamma processing in the visual cortex during pulvinar activation/deactivation.

In our study, alpha waves were less represented in deep cortical layers. This distribution of responses was independent of the effect of LPl inactivation (Figure 5A). Previous findings in awake monkeys have demonstrated that low-oscillation (beta waves) frequencies are more represented in infragranular layers [11]. The source of such divergence can come by the different distribution of ionotropic receptors that the visual cortexes of monkeys and cats have. A candidate for the facilitation of alpha waves is the N-methyl-D-aspartate receptors (NMDAr), which have slow activation kinetics compared to the α-amino-3-hydroxy-5-methyl-4-isoxazolepropionic acid receptor (AMPAr) [29,42]. In monkeys, NMDAr are distributed mainly in layers II/III and V of early visual cortical areas. In cats, such receptors are located in supragranular (layers II/III) layers of areas 17, 18 and 19 [43]. The distribution of NMDAr in area 21a is unknown. The anatomical and functional correspondence of alpha oscillations, given the distribution of NMDA receptors, emphasizes the importance of further analyzing the visual cortex of cats as a model for the propagation of visual activity. 

### 4.2. Generation of Gamma Waves

During LPl inactivation, oscillatory responses in layer IV of area 21a were dominated by gamma waves. This gamma-band frequency increase is the most prominent and consistent result of the present study. The mechanism by which gamma waves are evoked during LPl inactivation remains a mystery. Gamma synchronization is evoked by the fast succession of excitatory (E) and inhibitory (I) neuronal responses [13], and hence, depends on the balance between E–I interactions. Furthermore, theoretical simulations have suggested that E–I synapses producing fast rhythms are located close to the soma [44]. A candidate for the inhibitory contribution to generate gamma rhythms are the parvalbumin-positive (PV) fast-spiking interneurons [45]. LP and cortical neurons may modulate gamma-range oscillations by targeting such PV cells. For the thalamic contribution, this prediction is supported by the projection of pulvinar neurons to inhibitory interneurons in the visual cortex of mice [46]. The localization of these pulvinocortical synapses can also be deduced by the distribution of different receptors located across cortical layers. One candidate of gamma-wave generators is AMPAr, given the fast kinetics that these receptors possess. In the rat neocortex, excitatory postsynaptic potential (EPSP) onto fast spiking inhibitory neurons is mediated by AMPAr containing GluR1 and GluR4 [47]. In cats, GABAergic neurons with GluR1 are more prevalent across layers II-VI of visual areas 17, 18 and 19, suggesting that the fast action of these receptors should participate in the generation of gamma oscillations [43]. Thus, based on previous and the current findings, one may hypothesize that pulvinocortical projections may be targeting such receptors [42,48]. Nonetheless, the distribution of AMPAr in area 21a and the position of synaptic contacts of pulvinocortical projections onto inhibitory neurons is unknown. Future anatomical studies would provide helpful insights into the mechanism generating gamma-band synchronization in layer IV by the afferent combination of cortical and pulvinar connections. 

Gamma-range oscillations in area V4 of visual cortex of primates have been reported to decrease when the pulvinar was injected with muscimol, a GABA_A_ agonist [7]. Contrary to our findings, the majority of gamma-oscillatory patterns in area 21a were high during periods of GABA injection in the LPl. One possibility to explain these divergences is that pulvinocortical projections change the balanced state between excitatory-inhibitory of area 21a, increasing the output of neuronal responses [9]. Another possibility is that the GABA injected in the LPl leads to a net change that excites the neuronal population, shutting inhibitory neurons that are largely expressed in the cat thalamus [49,50]. In agreement with our findings, pulvinar deactivation by GABA in area V2 of primates yields changes in stimulus-driven responses [51]. Together, our findings and previous studies in primates demonstrate that the cortical activity is profoundly affected by the inactivation of the pulvinar, suggesting that the transthalamic pathway is essential for the control of the generation of gamma waves in the cortical processing of visual information. 

### 4.3. Mechanism of Action of Pulvinar-Cortical Projections

**Figure 7:**
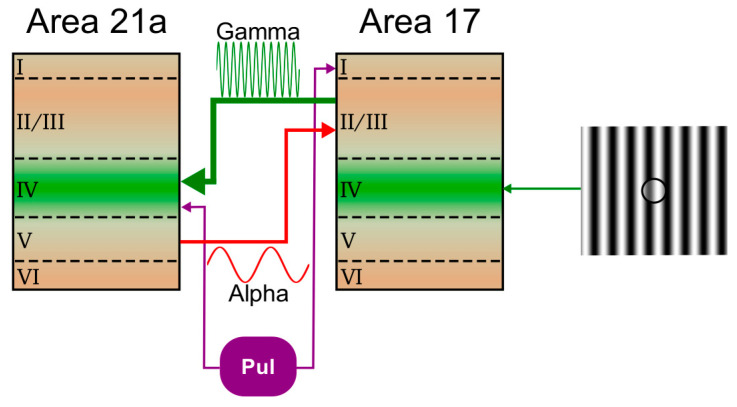
LPl control of feedforward and feedback processing in the early visual cortex system of cats. Putative wiring diagram for the frequency and layer-specific feedforward and feedback processing in which the area 17 receptive field is stimulated with a sinusoidal drifting grating. The LPl inactivation resulted in increased neuronal synchronization in the gamma band promoting a feedforward drive from area 17 to 21a (thick green line). Feedback coupling from area 21a to area 17 in the alpha band (thick blue line) is also increased during the pulvinar inactivation. The feedback processing may be mediated by NMDA receptors. The pulvinar could synchronize selectively neurons from area 17 to 21a to attend a visual stimulus, choosing the relevant feedback route to amplify the feedforward path.

Our current experimental results suggest that the LP is involved in the transmission of information by gating alpha and gamma waves throughout feedback and feedforward cortical pathways, respectively (Figure 7). Effective LP projections seem to have more driver influences toward area 21a, given the large amplification of gamma waves in layer IV during periods of GABA injection. Similar results were observed in the CRF of neurons in area 21a, in which the LPl inactivation generated an increase in the curve dynamic range [9]. Our results also show that feedback signals were more predominant at low oscillations in supragranular layers. Similar results have been found in the visual cortex of awake primates performing attentional-related tasks [12,39]. In that study, recordings in areas V1 and V4 indicated the occurrence of large alpha-wave feedback signals in superficial layers of V1, whereas gamma-band responses were elicited in layer IV, i.e., representing the feedforward pathway toward area V4. Theoretical works seem to validate our results, given that, in the simulations, the pulvinar facilitates the feedforward gamma transmission between two cortical areas [52,53]. Further works associating low and high oscillatory LFP signals may help to understand the nature of bottom-up and top-down pathways. Nonetheless, our findings suggest that the pulvinar should be considered as the gate of propagation of sensory information toward lower and higher cortical areas. Thus, one may hypothesize that the pulvinar is essential for an efficient feedforward and feedback information flow in the visual cortex.

In our study, the LP inactivation influenced the feedback alpha band evoked before the stimulus onset. This alpha activation is similar to the delay period of attentional-related tasks. As reported in previous works, the increase in alpha range is followed by the increase in gamma waves during the visual stimulation [54]. Thus, pulvinar–cortical projections may be the source either of the top-down influences in areas 17 and 18 or bottom-up for higher cortical areas [12,13,42]. We are aware that one has to be cautious in discussing attention-related gamma-band processing in experiments performed on anesthetized cats. Anesthesia is playing an essential role in the dynamic change of oscillatory activities. For example, before or after the inactivation of the thalamus, the gamma-band frequency is similar to baseline levels. Since halothane evokes spontaneous oscillations [37], the visual responses evoked are reduced when they are normalized by baseline periods. Anesthesia may also explain why the feedforward coupling (in the GC analysis) between areas 17 and 21a is limited to 30 Hz [41].

Nevertheless, our results support that the microcircuitry of the transthalamic pathway targeted here is responsible for similar changes observed in awake animals. Recent work has shown that the prefrontal cortex has direct functional connections with the visual cortex [55]. The oscillatory nature of these thalamocortical interactions may explain why schizophrenic patients have both disrupted gamma rhythms [56] and structural changes (reduced volume and cell numbers) in the pulvinar [57].

## Supplementary Materials

The following are available online at https://www.mdpi.com/2411-5150/4/2/22/s1, Figure S1: Validation of GABA injection in LP nucleus, Figure S2: Influence of MUA activity on the oscillatory cortical responses of areas 17 and 21a across cortical layers, Figure S3: The coupling index was calculated for the functional feedback connection between layer VI of area 21a and layer II/III of area 17, Table S1: Effect of LPl inactivation on the MUA of areas 17 and 21a, Table S2: Correlation between MUA activity on the oscillatory cortical responses of areas 17 and 21a across cortical layers, Table S3: Effect of LPl inactivation on alpha- and gamma waves of areas 17 and 21a, Table S4: The oscillatory activity (db from baseline/s) and %Var(Ctr) for alpha- and gamma waves of areas 17 and 21a across conditions, Table S5: Effect of LPl inactivation on alpha- and gamma waves across layers of areas 17 and 21a, Table S6: The oscillatory activity (db from baseline/s) and %Var(Ctr) for alpha- and gamma waves of areas 17 and 21a across cortical layers.
